# Supplementary material for: Direct imaging of residual oxygen disorder in an infinite-layer nickelate superlattice via multislice ptychography
Source: Nat Commun. 2025 Dec 11;16:11076. doi: 10.1038/s41467-025-67124-6 (PMC12698712; doi:10.1038/s41467-025-67124-6)
Supplement: Supplementary file 1 — Supplementary Information [file 41467_2025_67124_MOESM1_ESM.pdf]

Supplementary information for
Direct imaging of residual oxygen disorder in an infinite-layer nickelate superlattice via multislice ptychography

Chao Yang[1]*, Hongguang Wang[1]*, Roberto A. Ortiz[1], Kelvin Anggara[1], Eva Benckiser[1], Bernhard Keimer[1], Peter A. van Aken[1]

[1]Max Planck Institute for Solid State Research, Stuttgart, 70569, Germany

*Corresponding authors: c.yang@fkf.mpg.de; hgwang@fkf.mpg.de

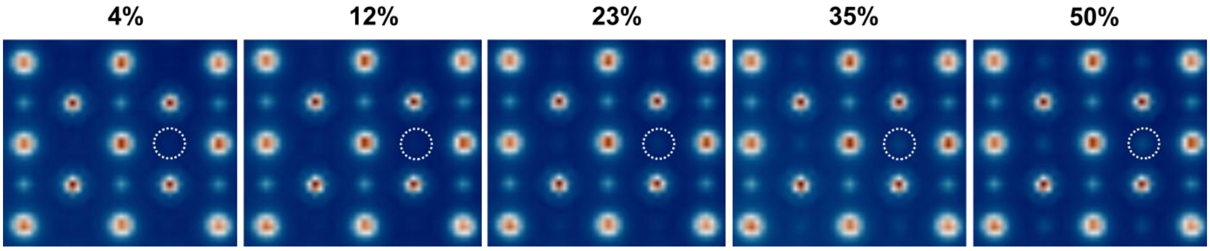

Figure S1. Simulated phase contrast images by multislice ptychography reconstruction with different oxygen occupancy (4%, 12%, 23%, 35%, and 50%) at the apical sites. The white circles mark the apical oxygen sites.

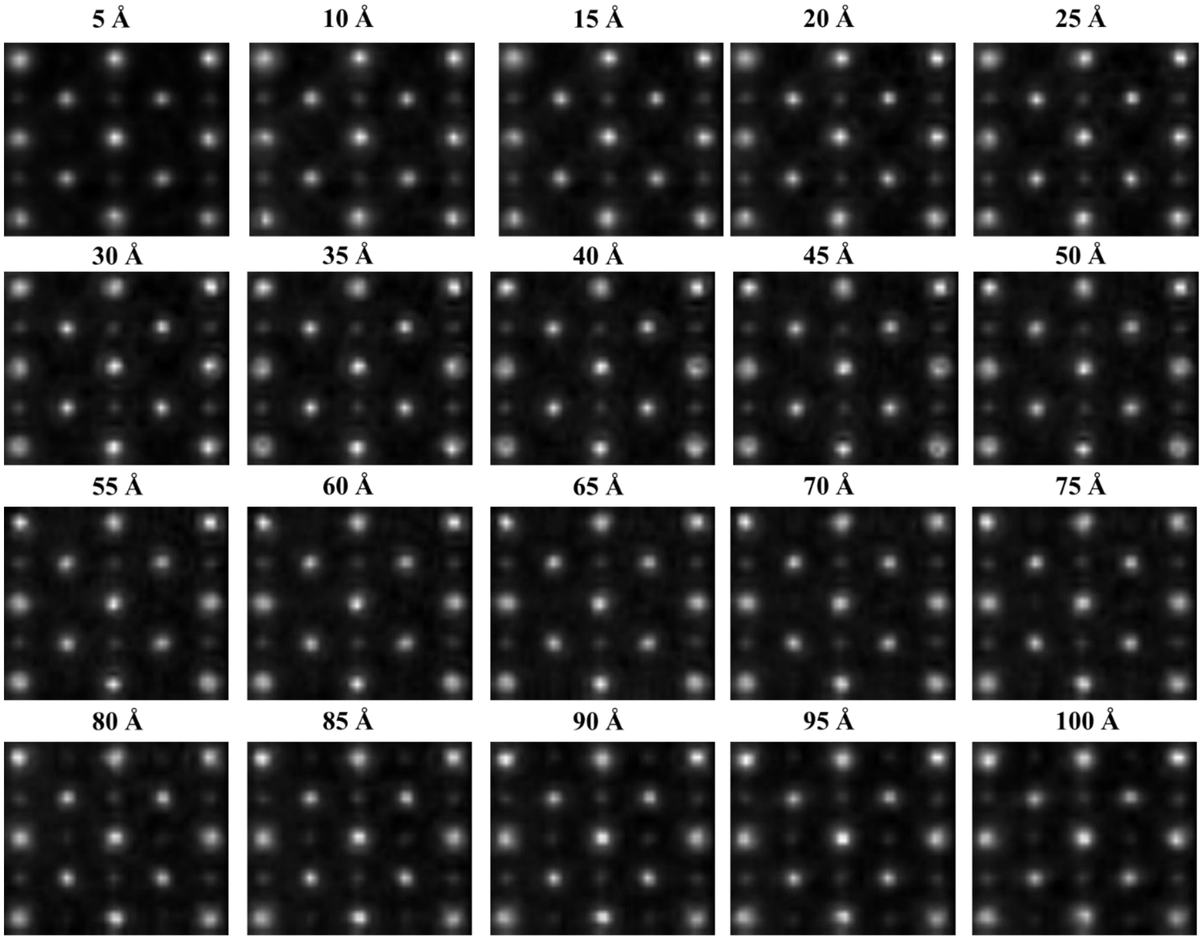

Figure S2. Simulated phase contrast images of $NdNiO_{2+x}$ with 23% occupancy of apical oxygen, showing the slices at different depths.

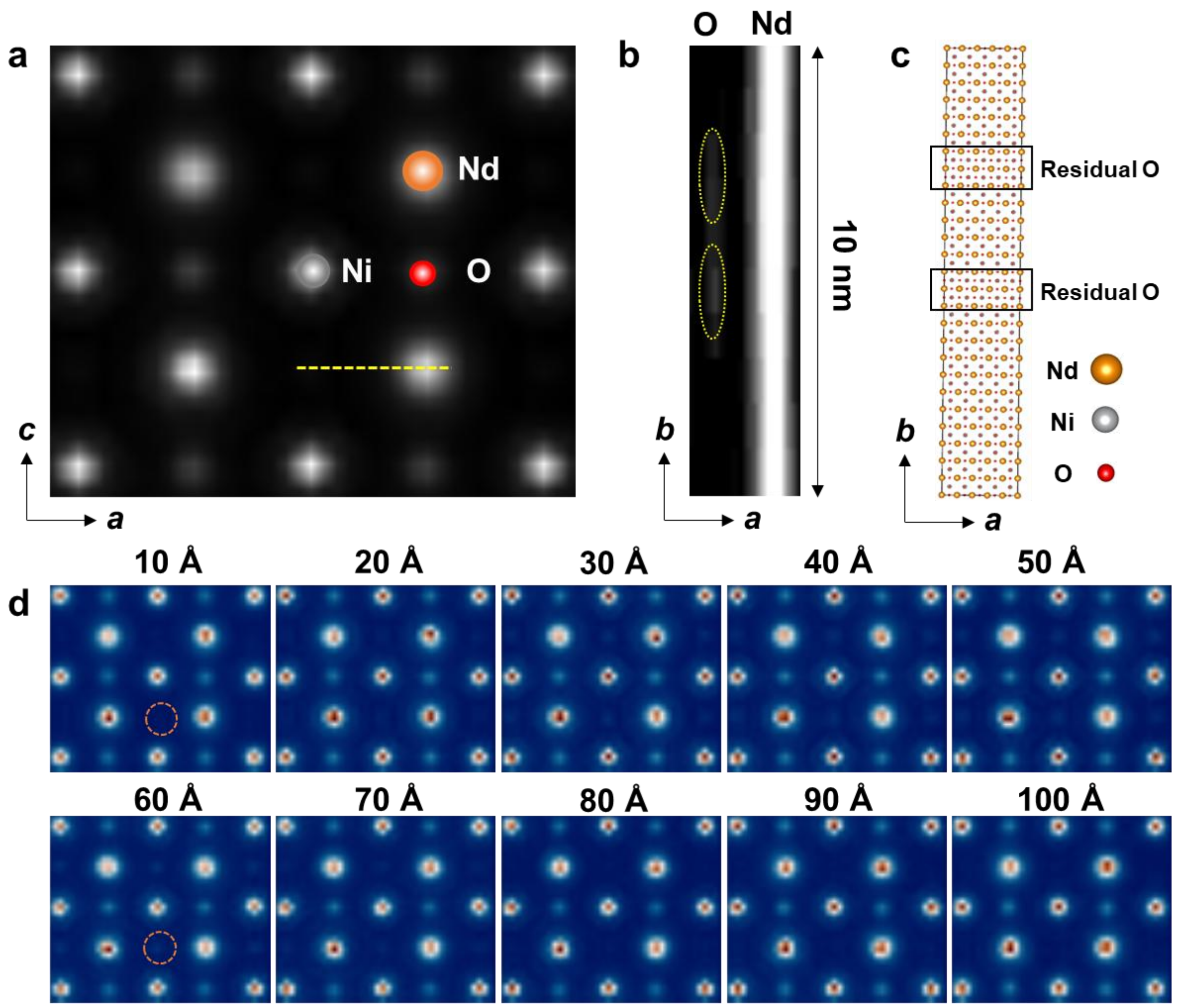

Figure S3. (a) A projected phase contrast image of a 10-nm-thick $NdNiO_{2+x}$ structural model. (b) The depth profile plot for oxygen and Nd atoms extracted from the yellow dashed line in (a). The elliptical dashed shapes mark the residual apical oxygen in the depth direction. (c) The corresponding structural model with the distribution of the residual apical oxygen. (d) Reconstructed phase contrast images at different depths.

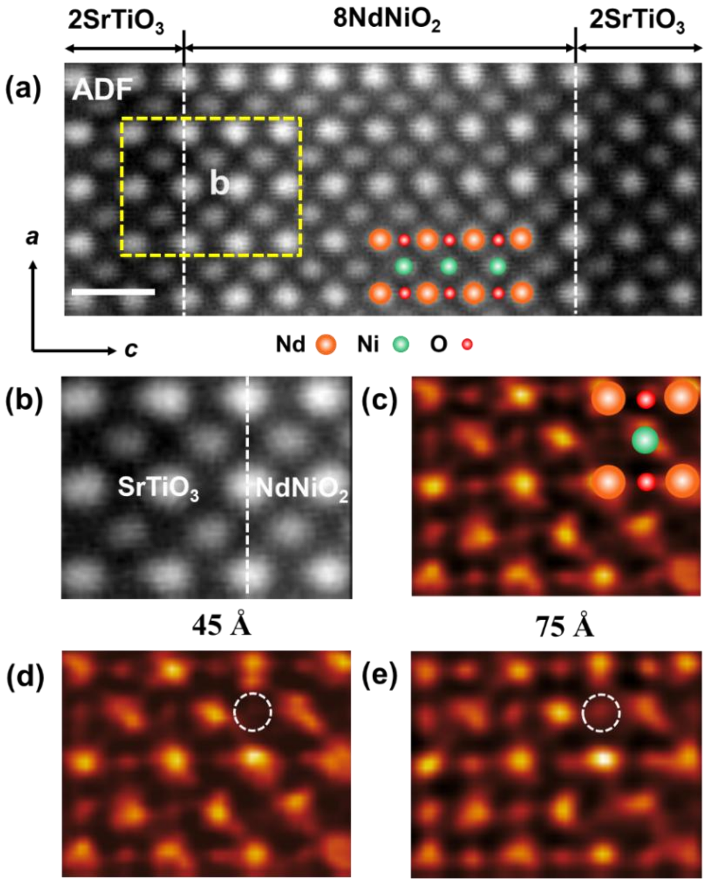

Figure S4. (a) Reconstructed annular dark field (ADF) image obtained from the 4D-STEM dataset, providing the atomic structure of the $8NdNiO_2/2SrTiO_3$ superlattice sample. (b) A magnified ADF image of the $SrTiO_3/NdNiO_2$ interface region, outlined by the yellow dashed box in (a). (c) The corresponding projected phase-contrast image reconstructed using multi-slice ptychography. Phase-contrast images at depths of (d) 45 Å and (e) 75 Å. The white dashed circles indicate the location of the residual apical oxygen.

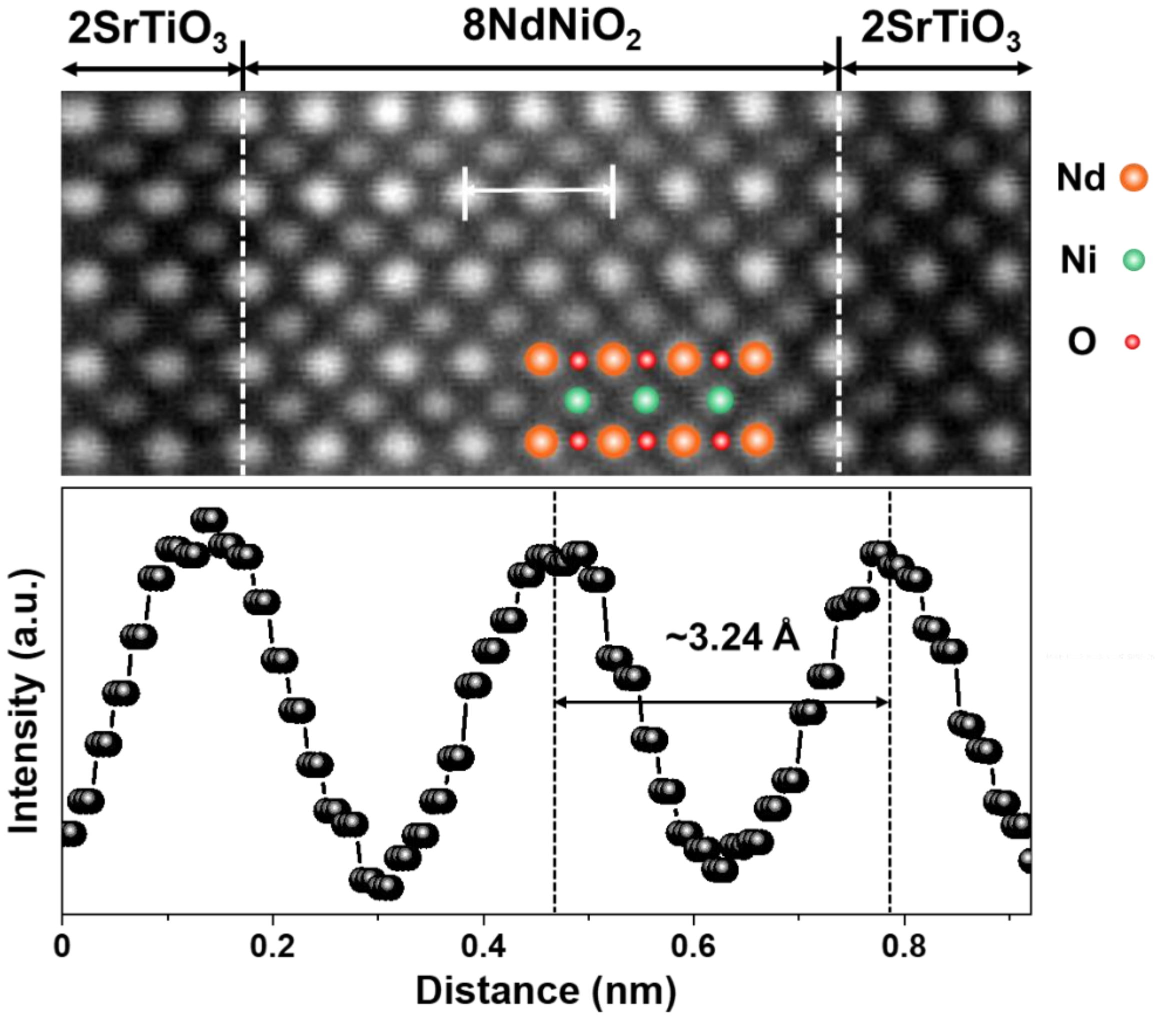

Figure S5. The HAADF image of the $8NdNiO_2/2SrTiO_3$ superlattice sample and the out-of-plane lattice spacing of the infinite layer nickelate. The line profile is extracted from the white line in the HAADF image.

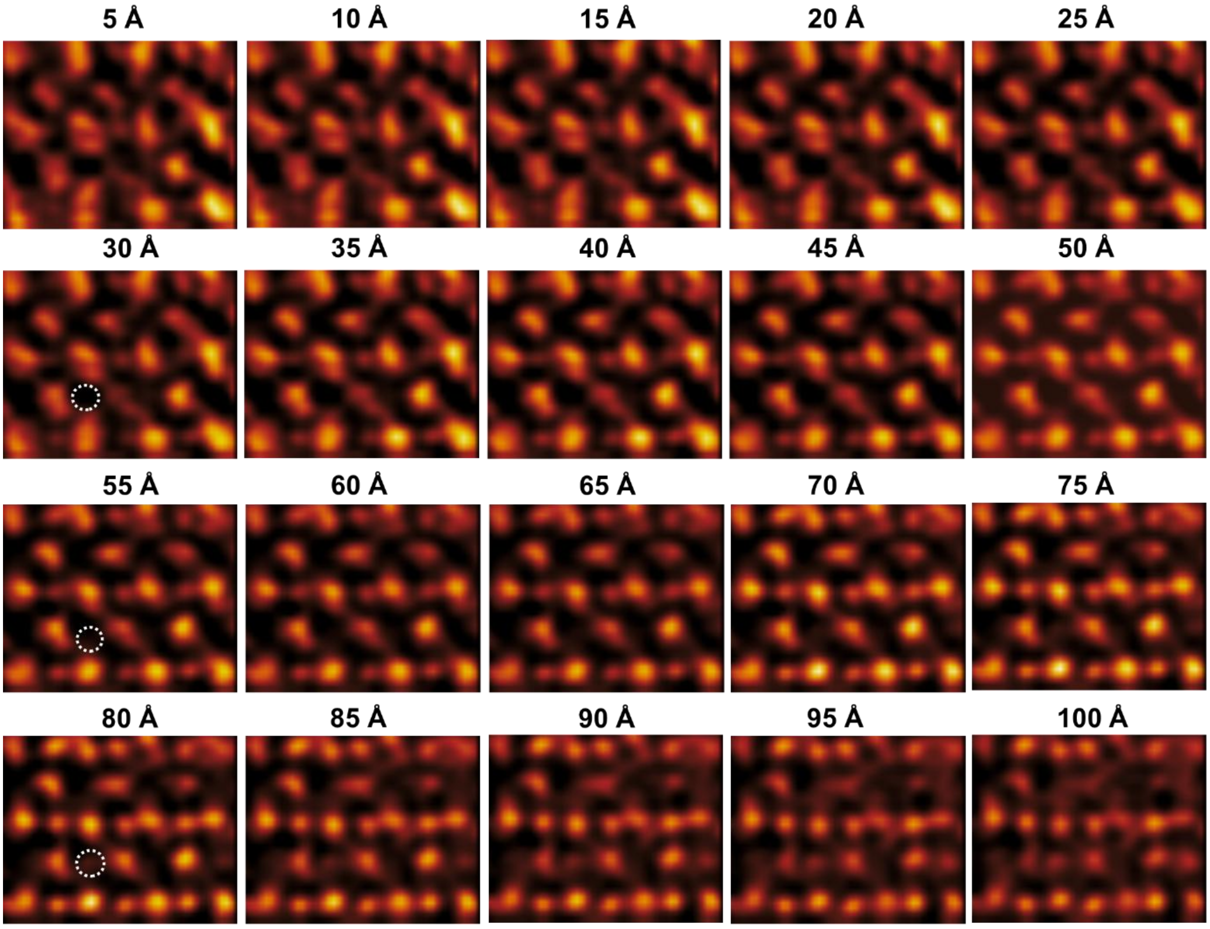

Figure S6. Experimental phase contrast images of $NdNiO_{2+x}$ showing all slices in different depths. The white circle shows the evolution of the apical oxygen contrast in different depths.

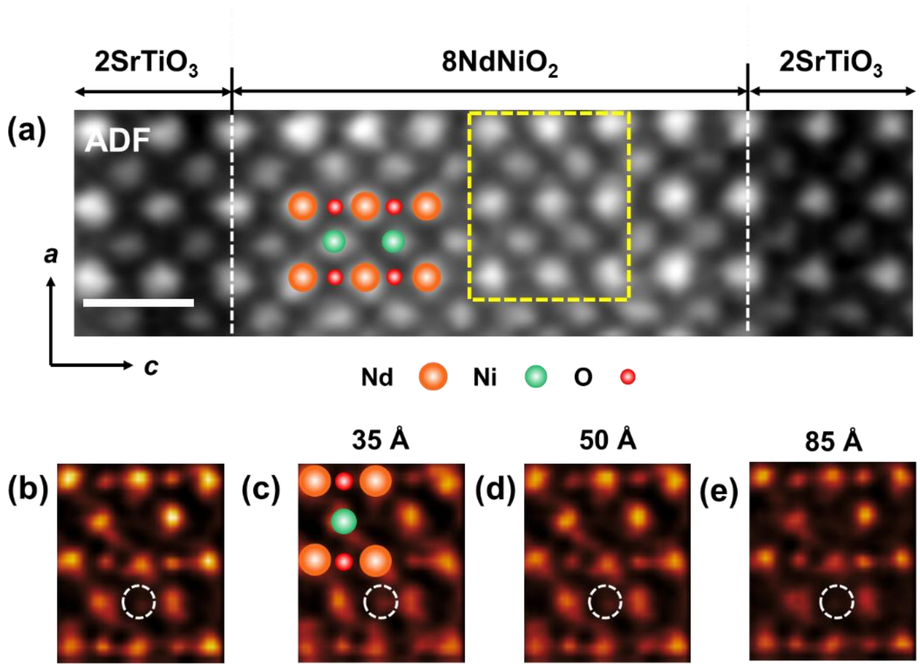

Figure S7. (a) Reconstructed annular dark field (ADF) image obtained from the 4D-STEM dataset, providing the atomic structure of the $8NdNiO_2/2SrTiO_3$ superlattice sample. (b) The projected phase-contrast image of the $NdNiO_2$ inner layer region were reconstructed using multi-slice ptychography, and are outlined by the yellow dashed box in (a). Phase-contrast images at depths of (c) 35 Å, (d) 50 Å, and (e) 85 Å. The white dashed circles indicate the location of the residual apical oxygen.

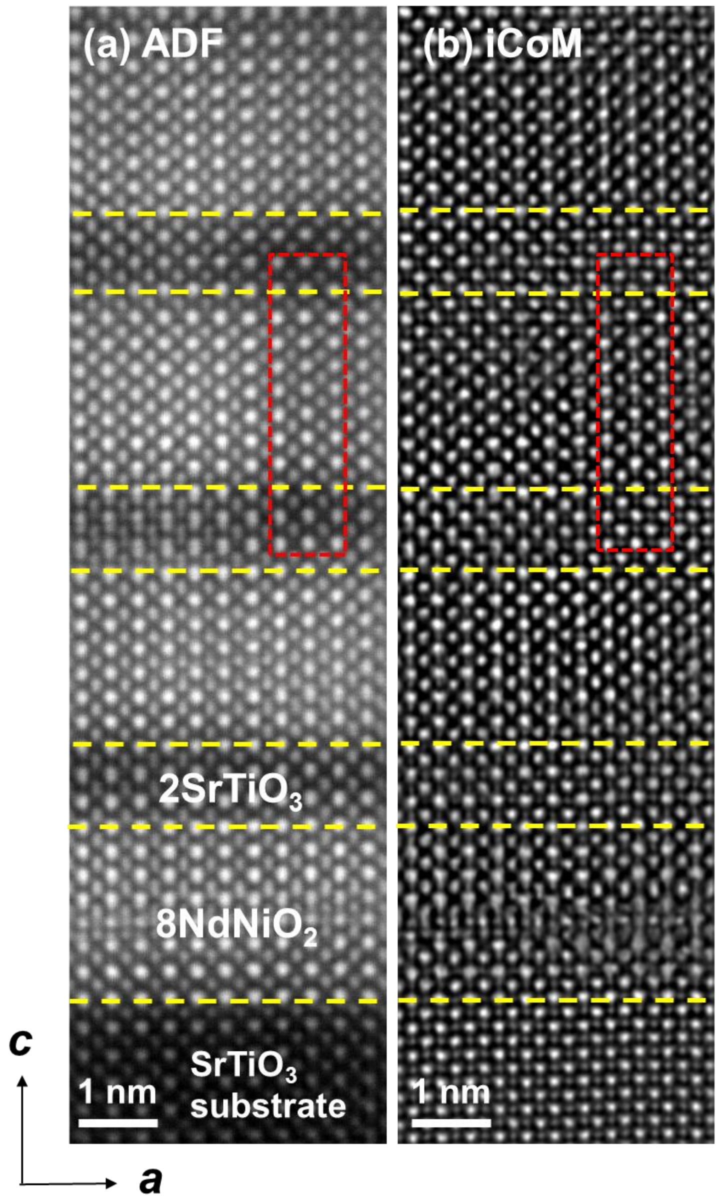

Figure S8. (a) The ADF image (b) iCoM image of an $8NdNiO_2/2SrTiO_3$ superlattice film grown on a $SrTiO_3$ substrate. The yellow dashed lines mark the $8NdNiO_2/2SrTiO_3$ interface. The red dashed boxes indicate the regions that have been cropped for Figure 2a.

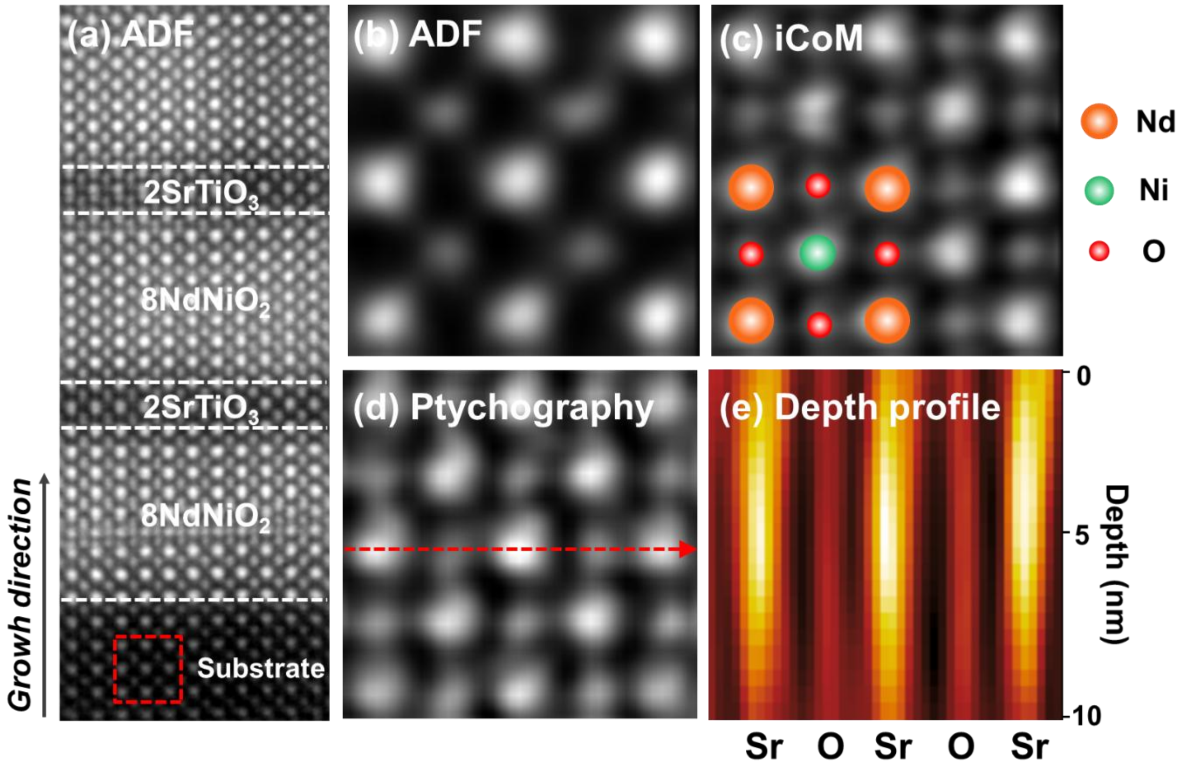

Figure S9. (a) The ADF image of an $8NdNiO_2/2SrTiO_3$ superlattice film growth on a $SrTiO_3$ substrate. Enlarged (b) ADF, (c) iCoM, and (d) multislice ptychography images of the $SrTiO_3$ substrate from the region marked by the red dashed box in (a). (e) Depth profile of Sr and O columns extracted along the red dashed arrow in (d).

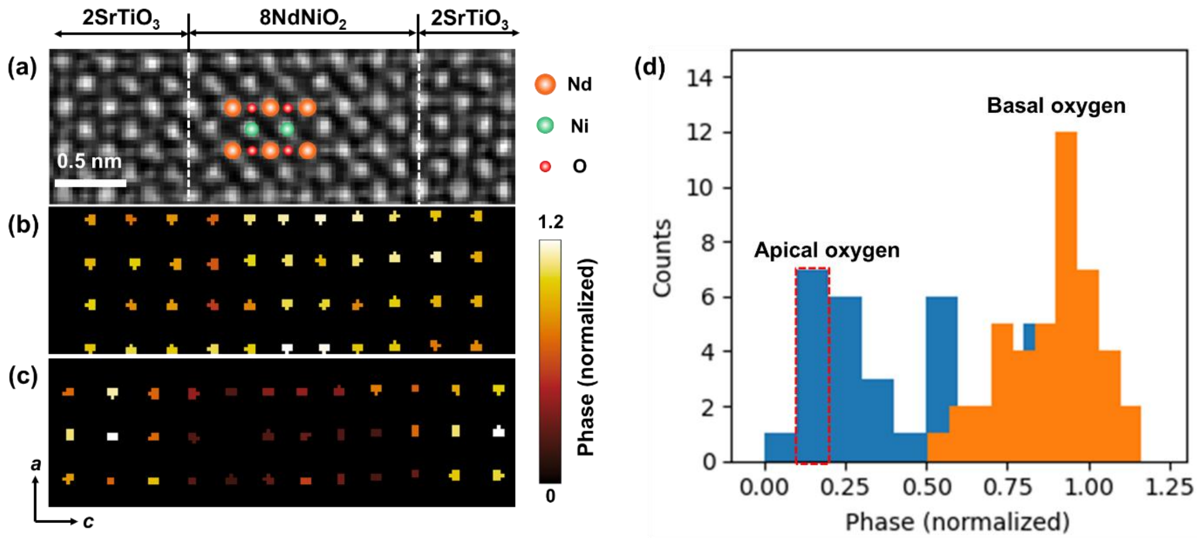

Figure S10. (a) Projected phase-contrast image reconstructed using multi-slice ptychography. The normalized phase contrast map of (b) the basal oxygen and (c) the apical oxygen extracted from (a). (d) Histogram of phases from basal and apical oxygen corresponding to (b) and (c), respectively. The oxygen sites in $SrTiO_3$ layer are considered fully occupied and can serve as the reference for 100% occupancy. The minimum observed phase contrast corresponds to 0% oxygen occupancy.

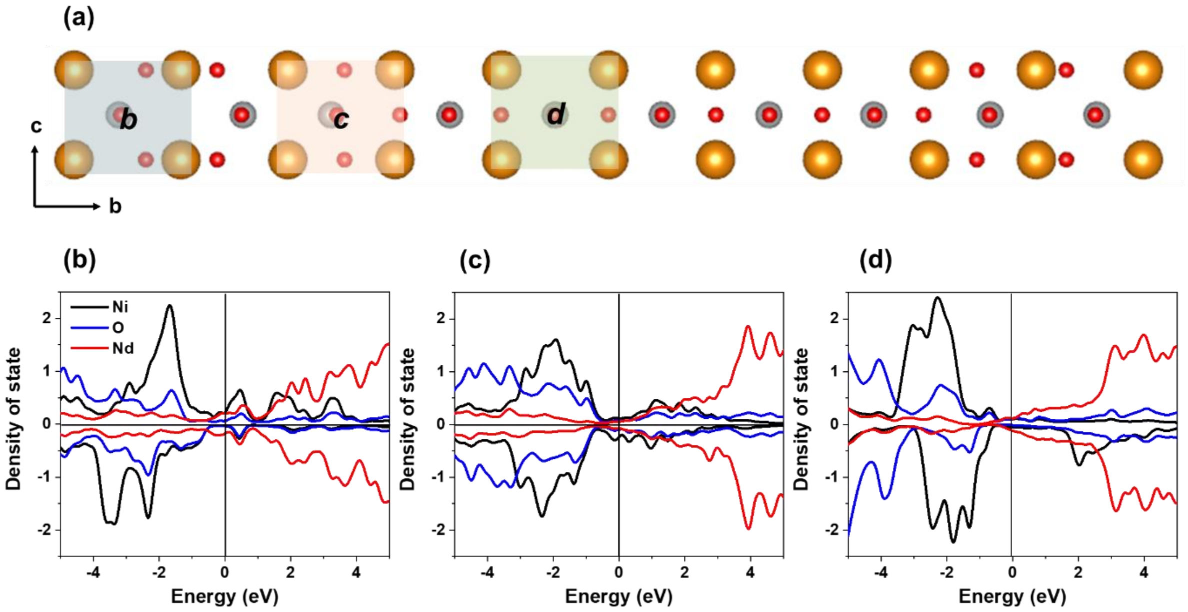

Figure S11. (a) Supercell model of the infinite-layer structure with two distinct domain orientations, *b* (*a*-axis) and *d* (*c*-axis), and their interface *c*. (b–d) Corresponding density of states (DOS) projections for the *a*-axis domain (b), domain interface (c), and *c*-axis domain (d).

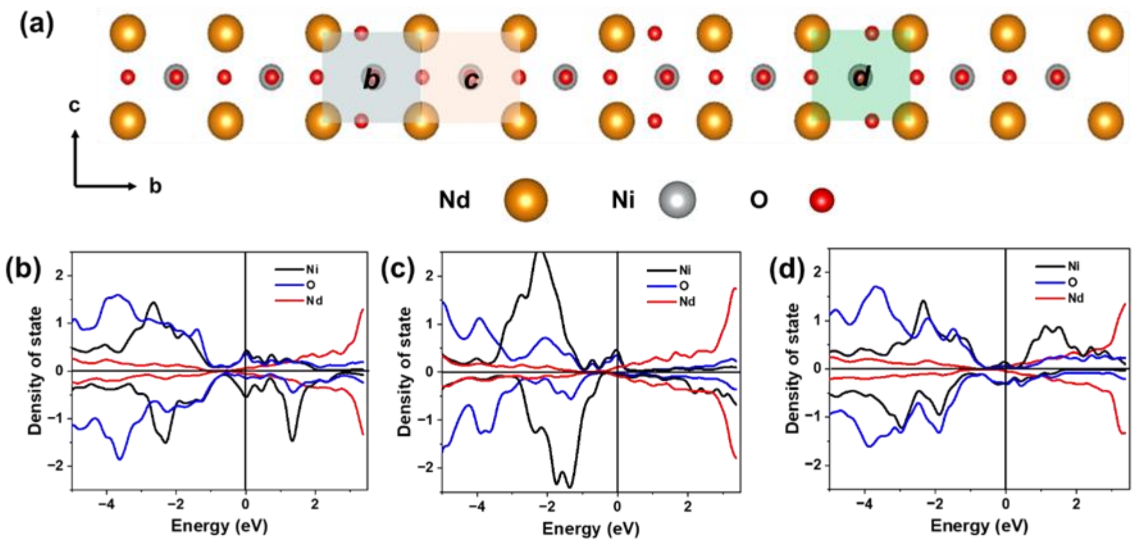

Figure S12. (a) Supercell model comprising the infinite-layer structure (c) together with the disordered residual perovskite units (b, d). The corresponding density of states is presented for panels (b), (c), and (d).

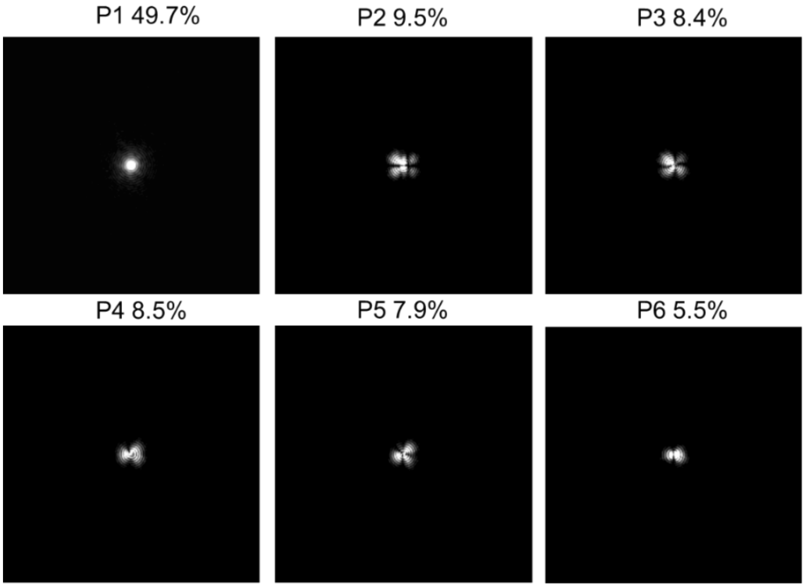

Figure S13. Intensity of the probe modes from the mixed-state algorithm. Mode indices and the corresponding fractional intensity of the total incident beam are labeled on the probe intensity images.
